# Valorization of Invasive Aquatic Weed (*Eichhornia crassipes*) Into a Sustainable Carbon Source in GIFT‐Based Biofloc Systems

**DOI:** 10.1155/anu/8838482

**Published:** 2025-12-11

**Authors:** Megha Lovejan, K. M. Mujeeb Rahiman, C. G. Joshy, R. Vipina, Remy Ntakirutimana

**Affiliations:** ^1^ School of Industrial Fisheries, Cochin University of Science and Technology, Kochi, Kerala, India, cusat.ac.in; ^2^ Fish Processing Division, ICAR-Central Institute of Fisheries Technology, Kochi, Kerala, India, cift.res.in; ^3^ PG Department of Microbiology, Majlis Arts and Science College, Malappuram, Kerala, India; ^4^ Center for Research in Natural and Environmental Sciences (CRSNE), University of Burundi, Bujumbura, Burundi, ub.edu.bi

**Keywords:** biofloc technology, carbon sources, *E. crassipes*, floc parameters, GIFT tilapia, sustainable farming

## Abstract

Biofloc technology (BFT) has emerged as a sustainable aquaculture strategy, enabling high productivity with minimal water and land use. While diverse carbon sources are employed in BFT systems, identifying cost‐effective and locally available alternatives can enhance profitability and adoption among farmers. This study evaluated the efficiency of five carbon sources, rice water (T1), jaggery (T2), sugar (T3), *Eichhornia crassipes* (T4), and a mixture of *E. crassipes* and jaggery (T5), compared to a control (CN) group without biofloc. Genetically improved farmed tilapia (GIFT) tilapia fry (10 fish per tank) were cultured for 60 days in triplicate tanks under controlled indoor conditions. After 15 days, all BFT treatments showed significantly higher weight gain than the CN (*p*  < 0.05). Although T2 achieved the highest weight gain overall, the differences from T4 and T5 were not significant by Days 45 and 60 (*p*  > 0.05). No significant differences were observed among BFT treatments in specific growth rate (SGR), daily weight gain, feed conversion ratio (FCR), or survival rate. Water quality parameters remained within optimal ranges, although total alkalinity declined across treatments from Day 15 onwards. Significant variation (*p*  < 0.05) was found in ammonia, nitrite, and nitrate concentrations among treatments throughout the trial. Floc volume (FV) increased steadily, peaking at Day 60 in the order T2 > T3 > T4 > T5 > T1. Floc porosity in T1 declined from 99.8% to 97.73%. Total solids were highest in T4 and T5. Bacterial counts were significantly greater in all BFT treatments compared to the CN (*p*  < 0.05). The results demonstrate that *E. crassipes*, alone or combined with jaggery, is an effective carbon source in BFT systems, supporting robust growth, microbial activity, and water quality. This approach offers an eco‐friendly, low‐cost strategy for improving the sustainability and economic viability of tilapia aquaculture.

## 1. Introduction

Aquaculture has become one of the fastest‐growing sources of animal protein globally and has surpassed wild‐capture fisheries as the dominant contributor to global seafood production [1]. In 2021, the aquaculture sector generated an estimated USD 296.5 billion in global production value, highlighting its critical role in the global economy [1, 2]. With the world’s population increasing, aquaculture is expected to play an even more pivotal role in combating food insecurity and malnutrition in the coming decades [3–6]. As eco‐efficient and nutrient‐rich food source, sustainable aquaculture systems offer considerable potential to enhance public health, support livelihoods, and promote environmental well‐being [4–6]. However, the expansion of aquaculture is constrained by significant challenges such as overexploitation of natural resources, environmental degradation, and poor governance [7], with the magnitude of these impacts varying depending on production methods, species farmed, and regulatory frameworks [5, 7].

Biofloc technology (BFT) has emerged as an innovative approach to mitigate several of these challenges. It enables high‐density fish production with minimal water exchange, reduced feed inputs, and improved nutrient recycling [8]. BFT relies on the addition of an external carbon source to manipulate the carbon‐to‐nitrogen (C:N) ratio in the culture water, stimulating the growth of heterotrophic bacteria that assimilate toxic nitrogenous compounds and convert them into microbial biomass [9–11]. This biomass, or “floc,” serves as a supplementary protein‐rich feed for aquatic animals, enhancing their growth while reducing feed wastage [12, 13]. Alongside carbon choice and C:N control (CN), operational management influences BFT performance. In tilapia systems, more frequent sludge removal reduced contaminant bioaccumulation, stabilized water quality, and improved growth, highlighting the need to manage solids in parallel with carbon dosing [14]. The process is effective even under zero‐water exchange conditions and can regenerate within hours under optimal aeration [15]. The efficiency of a BFT system depends heavily on the type of carbon source used [16]. Microorganisms depend on carbohydrates as their primary energy source, and the digestibility and composition of the carbon source greatly influence microbial growth, floc structure, and the physiological responses of farmed species [17]. The choice of carbon source also affects water quality, floc volume (FV), nutrient profile, and the retention of bioactive compounds such as extracellular enzymes and pigments [18–22]. Recent studies further underscore that the type and management of carbon inputs materially shape BFT outcomes in finfish. In Nile tilapia, molasses and tapioca flour yielded distinct profiles in water chemistry, growth, hematology, immune and antioxidant responses, confirming that carbon source selection is not interchangeable [23]. Complementary work in common carp shows that tapioca‐based biofloc under zero exchange improves growth and non‑specific immunity, reinforcing the utility of readily fermentable, low‑cost carbohydrates in heterotrophic systems [24]. Beyond growth, biofloc culture has been associated with enhanced hemato‑biochemical status and superior muscle composition compared with conventional pond systems, linking BFT to fish health and fillet quality [25]. Therefore, the use of accessible, cost‐effective, and nutritionally balanced carbon sources is essential to maximize the benefits of BFT while maintaining economic viability. Low‐cost agricultural and industrial by‐products such as jaggery, sugar, and molasses have shown promise in tilapia culture under BFT conditions [26–29]. Given their omnivorous feeding habits and high adaptability, tilapia are suitable candidates for intensive biofloc systems [30]. Together, these advances justify evaluating locally available, low‑cost carbon sources and practical husbandry CNs Water hyacinth (*Eichhornia crassipes*) is one of the world’s most invasive aquatic macrophytes, proliferating rapidly and severely disrupting aquatic ecosystems and human activities [31]. It spreads at an astonishing rate, producing 1.4 km^2^ of surface biomass and ~28,000 tonnes annually [30]. Its negative impacts include obstructing navigation, reducing biodiversity, promoting disease vectors, and degrading water quality [32, 33]. However, this nuisance weed contains a valuable nutrient profile, ~46.65% carbohydrates, 19.11% protein, 1.21% lipid, and 15.30% ash, making it a potential low‐cost bioresource for aquaculture applications [34]. Similarly, rice soup or rice water, a common household waste, contains high levels of carbohydrates (~76.7%) and can be repurposed as a fermentable carbon source [35, 36]. Despite the nutritional potential of these unconventional carbon sources, they remain underutilized in biofloc systems. In particular, the application of *E. crassipes*, either alone or in combination with jaggery and rice soup, has not yet been evaluated as a carbon input in BFT. This study aims to fill that gap by investigating the effects of different carbon sources, jaggery, sugar, rice water, *E. crassipes*, and a mixture of *E. crassipes* with jaggery, on water quality, floc development, bacterial load, and the growth and proximate composition of genetically improved farmed tilapia (GIFT) cultured in a biofloc environment. This approach also explores the valorization of biomass waste for circular aquaculture and sustainable food production.

## 2. Materials and Methods

### 2.1. Experimental Design

The experiment was conducted over a period of 60 days at the hatchery complex of the School of Industrial Fisheries, Cochin University of Science and Technology (CUSAT), Kerala, India. GIFT fry (*O. niloticus*) with an average initial weight of 1.57 ± 0.001 g were procured from the MPEDA‐RGCA Multispecies Aquaculture Centre (Vallarpadam, Ernakulam, Kerala) and acclimatized under laboratory conditions for 1 week. A total of 18 fiberglass‐reinforced plastic (FRP) tanks with a capacity of 100 L each were used. Tanks were disinfected with a 10 ppm potassium permanganate solution, rinsed, and filled with 70 L of dechlorinated freshwater. Aeration was applied for 5 days prior to fish stocking.

The experiment followed a completely randomized design (CRD) with six treatments in triplicate, each tank stocked with 10 fish, totaling 180 fries. The treatment groups were T1 (rice water), T2 (jaggery), T3 (sugar), T4 (*E. crassipes* powder), T5 (Mixture of *E. crassipes* powder and jaggery (1:1, w/w), and CN (clear water system without biofloc or carbon source). All tanks were maintained under indoor hatchery conditions with a 12:12 h light:dark photoperiod and continuous aeration provided through air stones connected to a centralized air pump. Ambient temperature was maintained between 28 and 30°C. Water lost to evaporation was replenished daily using dechlorinated freshwater with a siphon pipe, taking care not to disturb the biofloc layer. Alkalinity was maintained between 100–150 mg/L by adding NaHCO_3_ (~4.2 g per 70 L) to each tank whenever levels dropped below 80 mg/L, applied weekly or as needed [12]. Fish were fed a commercial floating pellet feed (Growfin, Growel Feeds Pvt. Ltd., India). The feed ingredients and proximate composition are presented in Table 1. Feeding was done three times daily at 5% body weight, adjusted biweekly based on group mean weights.

**Table 1:** Ingredients and proximate composition of the commercial feed and carbon sources used in this study.

Ingredients	Rice and rice products	Soybean meal	Maize and maize products	Fish meal	Fish oil	Soy lecithin	Vitamin premix^a^	Mineral premix^b^
Proportion (%)	30	30	25	10	1	1	Added	Added

	Proximate composition					—	—	—
	Feed	Rice water	Jaggery	Sugar	*E. crassipes*	—	—	—

Crude protein (%)	30	4.6	3.7	0	19.23	—	—	—
Crude fat (%)	5	2.6	0.5	0	1.2	—	—	—
Crude fiber (%)	5.5	0.2	1.2	0	18.4	—	—	—
Moisture (%)	12	—	—	—	—	—	—	—
Ash (%)	9	4.7	9	0.4	16.8	—	—	—
NFE^c^ (%)	—	87.9	85.6	99.6	44.37	—	—	—

^a^The premix product used contained vitamins such as A, B, C, D_3_, and K_3_.

^b^The premix product used contained minerals such as Ca, P, Na, Mg, Zn, Mn, Fe, Cu, Se, and Co.

^c^Carbohydrate (Nitrogen‐free extract), calculated by difference = 100 − (crude protein + lipid + ash + crude fiber).

### 2.2. Preparation of Carbon Sources

Freshwater hyacinth (*E. crassipes*) was collected from Vembanad Lake, Kerala, India. The plants were washed thoroughly with clean water to remove mud, insects, and debris. The leaves and petioles were chopped into smaller sections and shade‐dried for 4–5 days, followed by oven‐drying at 60°C for 24 h to ensure complete moisture removal. The dried biomass was then ground into fine powder using a mechanical grinder and stored in airtight containers at room temperature until use. Rice water was obtained by collecting the starchy water discarded during the first rinse and boiling of white rice in domestic kitchens. Approximately 1.5 L of rice water was collected per 500 g of rice cooked. The collected rice water was cooled, filtered through a fine mesh to remove solid residues, and used fresh. Commercial‐grade jaggery and sugar were procured from a local market in Kochi, Kerala. All carbon sources were handled under clean laboratory conditions to avoid microbial contamination before use.

### 2.3. Preparation of Biofloc Inoculum

Biofloc inoculum was prepared using a commercial probiotic solution Aqua Maple EM1 Aqua Magic (Reg. No. CAA/Jy17/PRO/00888). For each treatment, 250 mL of the EM1 inoculum was mixed with 4 L of dechlorinated water, and 250 g of the designated carbon source (rice water, jaggery, sugar, *E. crassipes* powder, or a 1:1 mixture of *E. crassipes* and jaggery) was dissolved in 1 L of water. The mixture was fermented in sterile, airtight plastic containers and incubated in dark conditions at ambient temperature (~28°C) for 7 days.

On the 0th day, 400 mL of the activated inoculum was added to each treatment tank. No inoculum or carbon source was added to the CN tanks. Continuous aeration ensured homogeneous suspension of flocs and maintained aerobic conditions favorable for heterotrophic microbial growth. To sustain a C:N ratio of ~15:1, the amount of carbon added daily was calculated based on feed nitrogen input, following the method of Avnimelech [37]. The estimated carbon contribution of each carbon source was based on its proximate composition, particularly carbohydrate and protein content (Table 1).

### 2.4. Water Quality Parameters

Water quality parameters, including temperature, pH, dissolved oxygen (DO), total alkalinity, total ammonia nitrogen (TAN), nitrite (NO_2_^−^), and nitrate (NO_3_^−^), were monitored throughout the 60‐day culture period. Temperature was measured daily using a standard mercury thermometer, while pH was recorded using a calibrated pH meter. DO levels were determined daily by the Winkler titration method. Total alkalinity was measured weekly following the standard procedure described by Bridgewater et al. [38]. Nitrogenous compounds, including TAN, nitrite‐N, and nitrate‐N, were measured weekly using a spectrophotometer. Absorbance readings were taken at 635 nm for TAN, and 543 nm for both nitrite‐N and nitrate‐N, as described by Bridgewater et al. [38]. FV was recorded weekly using a 1‐L Imhoff cone by allowing floc particles to settle undisturbed for 30 min and reading the settled volume in mL/L. In CN tanks, which lacked biofloc formation, siphoning was performed daily before feeding to remove waste, and 20% of the water was replaced daily to maintain water quality.

### 2.5. Growth Parameters

Prior to stocking, the initial length and weight of fry were recorded. Fishes were sampled at 15‐day intervals from each triplicate group to measure length and weight. Growth performance and feed utilization parameters, including total weight gain (TWG), specific growth rate (SGR), feed conversion ratio (FCR), protein efficiency ratio (PER), daily increment (DI), and survival (%), were calculated as follows:

• TWG (g) = final weight (g) − initial weight (g).
• SGR (%) = Ln (final weight) – Ln (Initial weight) × 100/number of days.
• FCR = dry feed intake (g)/fish live weight gain (g).
• PER = fish live weight gain (g)∕dry protein fed.
• DI (g/day) = (final weight − initial weight)/experimental days.
• Survival rate (%) = total number of fish harvested/total number of fish stocked × 100.

### 2.6. Floc Parameters

The floc morphology, such as color, size, and characteristics, was analyzed to identify the variations in the physical properties and overall efficiency of the biofloc systems. The morphological features were analyzed using Leica DM6 (DFC 450, German), and the floc size was measured in µm. The floc color was matched as per the standard color code chart [39]. Biofloc water was collected and left undisturbed for 20–30 min to allow the floc particles to settle down. The volume of settled floc was measured as FV in mL/L [15]. Floc porosity was calculated based on the ratio of the water volume to the volume of settled floc [40].

• Porosity (%) = (1–FV/WV) × 100

FV index (FVI) was obtained using FV and floc concentration [41]. It is calculated using the formula.

• FVI = FV (mL)/floc concentration (g).

Floc density index (FDI) was calculated using FVI, and it is the grams of floc that occupies a volume of 100 mL after 30 min of settling [42].

• FDI = 100/FVI.

**Figure 1:**
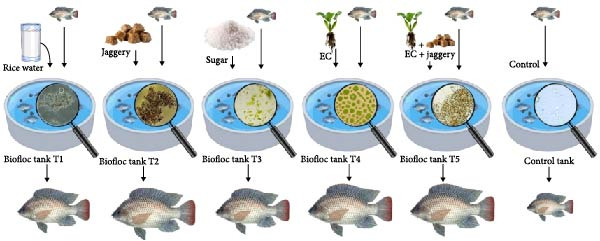
Overview of experimental biofloc treatments showing different carbon sources, tank conditions, floc appearance, and fish growth at the end of the 60‐day culture period.

The TSS, TDS, and TS levels were estimated at the end of the experiment by the gravimetric method [43]. A schematic overview of the experimental setup, including the carbon sources used in each biofloc treatment, is presented in Figure 1.

### 2.7. Bacterial Counts

Bacterial counts from biofloc and clear water samples were estimated by following the standard procedure [44]. The samples were taken during the 0th, 30th, and 60th days of the culture period using presterilized bottles, serial dilution was carried out, and 0.1 mL of the samples was plated on trypticase soy agar (TSA) in triplicate and incubated for 24–48 h at 37°C. After incubation, the bacterial colonies on the TSA plate were counted and expressed as colony‐forming unit (CFU/mL).

### 2.8. Proximate Composition

The proximate composition of fish and floc samples was determined using the standard method. The moisture content was determined by drying at 105°C, protein content was estimated using the Kjeldahl method, fat content was determined by the Soxhlet extraction method, and ash content was determined by incinerating preweighed samples in a muffle furnace at 600°C for 6 h.

### 2.9. Data Analysis

Two‐way analysis of variance (ANOVA) was performed to see the direct effect of treatment, culture days, and their interaction on water quality, growth, floc parameters, and bacterial count. The marginal means were compared using Tukey’s test, and pairwise comparisons of interaction effect on means were conducted using *t*‐test at 5% level of significance (*p*  < 0.05). One‐way ANOVA was performed to see the effect of treatment on proximate composition on the completion of the study. Tukey’s test was used to compare the treatment means as a post hoc analysis at 5% level of significance (*p*  < 0.05). All the analyses were done using SAS software version 9.3 (SAS/STAT 15.3).

## 3. Result

### 3.1. Water Quality Parameters

The water quality parameters during the 60‐day culture period are shown in Table 2. Overall, the mean temperature, pH, and DO were in the optimal range for a zero water exchange system. The temperature and pH in all treatments were ranged between 27.9–29.9 and 6.2–7.3, respectively, and exhibited a downward trend from the start of the experiment (Figure 2a,b). On the 30th day, the pH experienced substantial variations between treatments, with the lowest measured value being 6.2 in T5 (*p*  < 0.05). A decreasing trend was noticed in the DO from the start of the experiment; however, DO levels did not show any significant difference (*p*  > 0.05) (Figure 2c). Total alkalinity showed a decreasing trend in all treatments, particularly from the 15th day onwards; however, no significant differences (*p*  > 0.05) were observed among the biofloc treatments with the CN (Figure 2d).

Figure 2Water quality parameters of GIFT tilapia during a 60‐day culture period: (a) temperature, (b) pH, (c) DO, (d) total alkalinity, (e) ammonia, (f) nitrite, and (g) nitrate.

**Figure:**
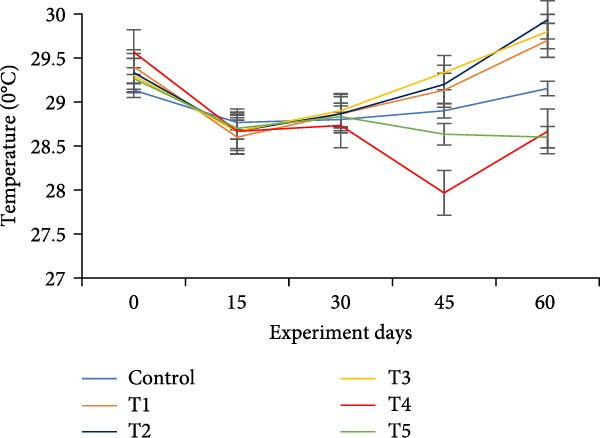
(a)

**Figure:**
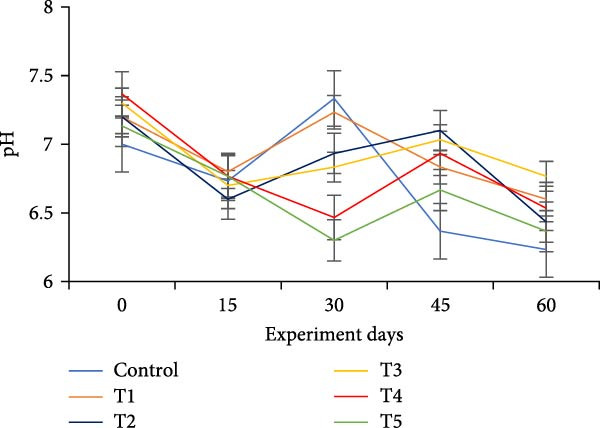
(b)

**Figure:**
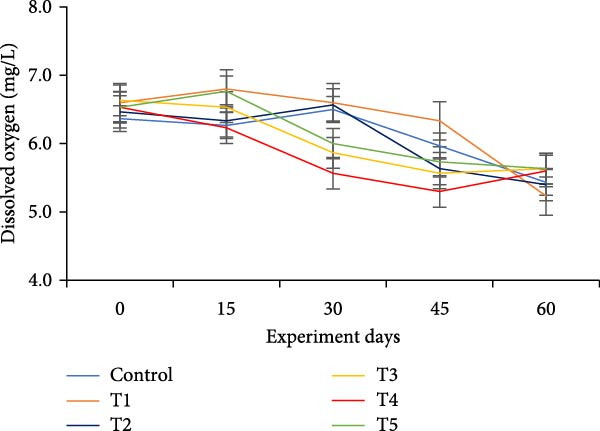
(c)

**Figure:**
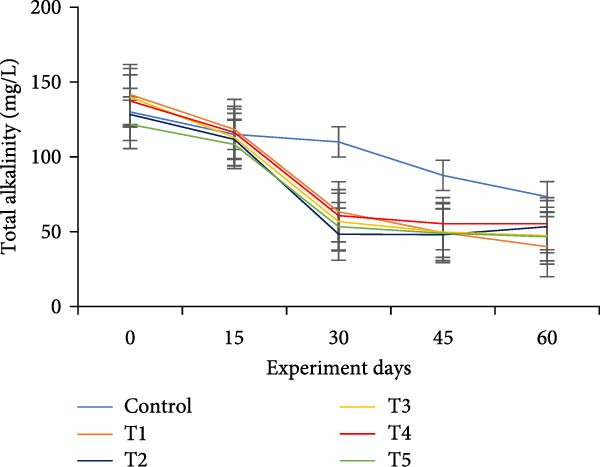
(d)

**Figure:**
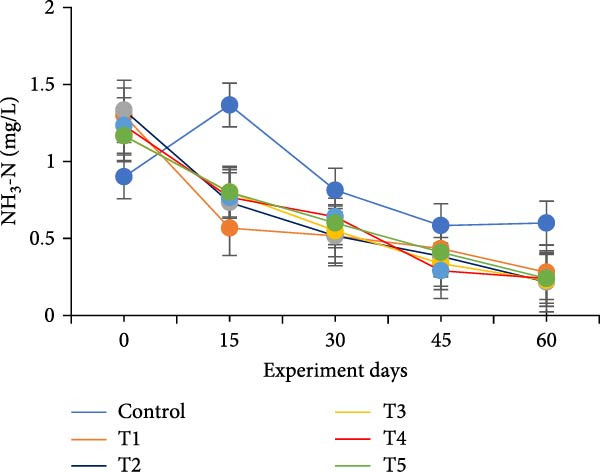
(e)

**Figure:**
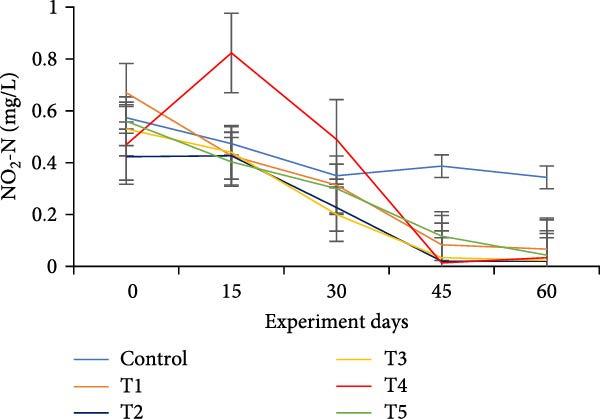
(f)

**Figure:**
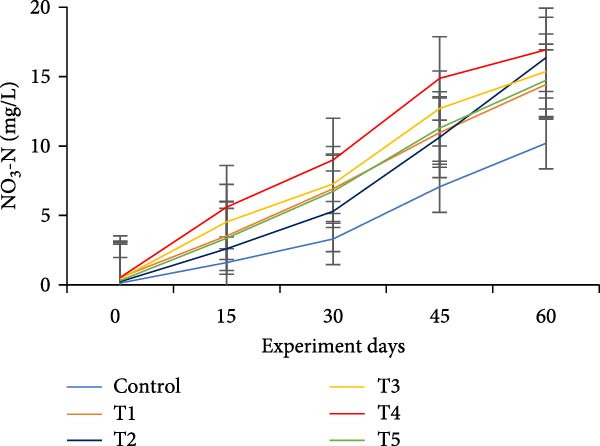
(g)

**Table 2:** Means of water quality parameters during the 60‐day culture period.

Parameter	T1	T2	T3	T4	T5	Control
*T* (°C)	29.14 ± 0.43^a^ (28.6–29.7)	29.2 ± 0.49^a^ (28.6–29.9)	29.2 ± 0.44^a^ (28.6–29.8)	28.72 ± 0.57^b^ (27.9–29.5)	28.80 ± 0.27^b^ (28.6–29.2)	28.76 ± 0.18^b^ (28.5–29.1)
pH	6.93 ± 0.27^a^ (6.60–7.20)	6.85 ± 0.33^ab^ (6.40–7.20)	6.92 ± 0.24^a^ (6.70–7.30)	6.81 ± 0.36^ab^ (6.20–7.25)	6.64 ± 0.34^ab^ (6.30–7.10)	6.73 ± 0.45^ab^ (6.20–7.30)
DO (mg/L)	6.31 ± 0.63^a^ (5.20–6.80)	6.08 ± 0.53^ab^ (6.40–5.40)	6.04 ± 0.50^ab^ (5.50–6.60)	5.84 ± 0.51^b^ (5.30–6.50)	6.13 ± 0.50^ab^ (5.63–6.70)	6.10 ± 0.42^ab^ (5.40–6.50)
Alkalinity (mg/L)	82.5 ± 3.31^ab^ (40.0–141.0)	77.9 ± 4.45^ab^ (48.0–128.0)	81.3 ± 3.58^ab^ (47.3–140.0)	85 ± 3.15^a^ (55.3–137.3)	75.8 ± 3.15^b^ (46.6–121.6)	103.2 ± 7.73^c^ (73.0–130.0)
TAN‐N (mg/L)	0.61 ± 0.40^b^ (0.20–1.30)	0.63 ± 0.43^c^ (0.20–1.10)	0.61 ± 0.38^b^ (0.20–1.16)	0.63 ± 0.40^b^ (0.24–1.23)	0.64 ± 0.36^b^ (0.20–1.10)	0.85 ± 0.32^a^ (0.50–1.36)
NO_2_‐N (mg/L)	0.31 ± 0.25^c^ (0.06–0.42)	0.22 ± 0.20^d^ (0.02–0.42)	0.24 ± 0.23^d^ (0.02–0.44)	0.36 ± 0.34^b^ (0.01–0.82)	0.28 ± 0.21^c^ (0.04–0.40)	0.42 ± 0.10^a^ (0.3–0.57)
NO_3_‐N (mg/L)	7.28 ± 5.5^c^ (0.53–10.96)	7.02 ± 6.5^c^ (0.23–14.4)	8.06 ± 6.04^b^ (0.43–16.3)	9.38 ± 6.7^a^ (0.53–16.9)	10.89 ± 5.8^c^ (0.32–26.3)	4.46 ± 4.1^d^ (0.13–10.2)

*Note:* Values (means ± SE) in the same row with different superscripts differ significantly (*p*  < 0.05) between treatments. The values in the parentheses show the observed ranges.

Significant differences (*p*  < 0.05) in NH_3_, NO_2_, and NO_3_ levels were observed among treatments throughout the culture period. In all tanks, including the CN, NH_3_, and NO_2_ concentrations showed a steady decline over the 60 days, except for a peak recorded in the CN and T4 tanks on the 15th day (Figure 2e–g). Furthermore, an increasing trend was noticed that the biofloc treatments consistently exhibited significantly higher NO_3_ levels compared to the CN (*p*  < 0.05).

### 3.2. Growth Parameters

During the initial 15 days, growth metrics such as length, weight, TWG, SGR, FCR, PER, and DI showed no significant variations (*p* > 0.05) among the treatments. However, after 15 days, substantial increases in fish weight were observed (*p*  < 0.05) in all the biofloc groups compared to the CN group. From the 30th day onward, T2 exhibited the highest weight, significantly (*p*  < 0.05) surpassing T4 and T5 (Figure 3a,b). Although T2 maintained the highest weight gain throughout the culture period, the difference from T4 and T5 was not significant (*p*  > 0.05) on 45th and 60th days. The CN group yielded the least with 17.19 ± 1.00 g. SGR and DI in all treatment groups increased significantly (*p*  < 0.05) with the culture period (Figure 3c,d). T2 had the highest DI among all biofloc treatments (*p*  < 0.05), except for T5, while the CN group had the lowest DI. T2 recorded the lowest FCR at 1.46 ± 0.45, which was significantly different from the CN groups, but not with the biofloc treatments (Figure 3e). T2 also had the highest PER (2.29 ± 0.03), which had no significant differences from T4, T5, and T1 (Figure 3f). By the end of the experiment, all biofloc treatments showed a higher survival rate than the CN group (Table 3). The highest survival rates were recorded in T2 and T3. However, no significant difference between BFT treatments was observed (*p*  > 0.05) at the end of the culture period. Biofloc treatments using *Eichhornia* yielded superior outcomes than widely used carbon sources and the traditional clear water systems.

Figure 3Growth parameters of GIFT tilapia cultured during a period of 60 days: (a) weight, (b) TWG, (c) SGR, (d) DI, (e) FCR, and (f) PER.

**Figure:**
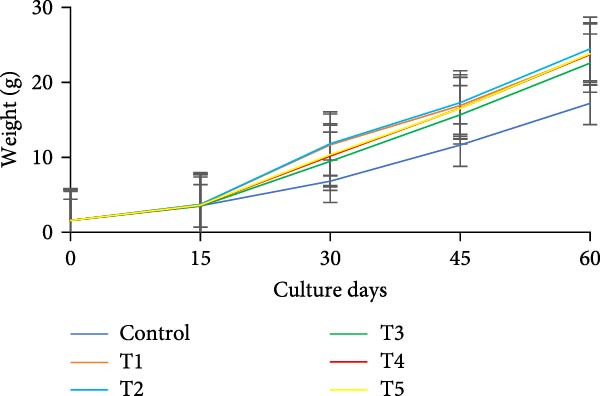
(a)

**Figure:**
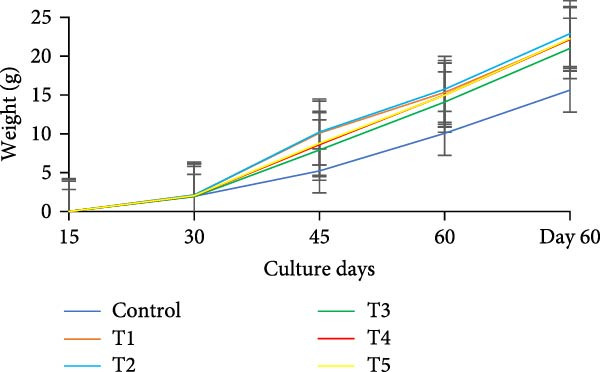
(b)

**Figure:**
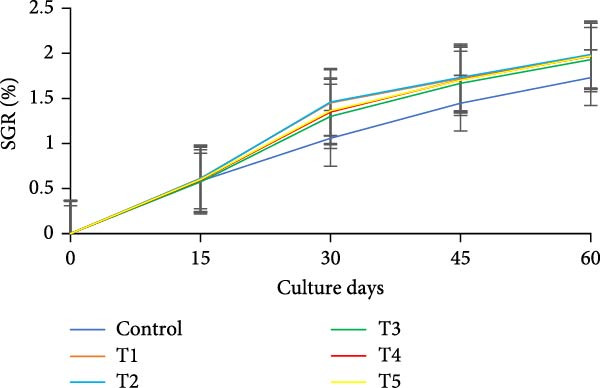
(c)

**Figure:**
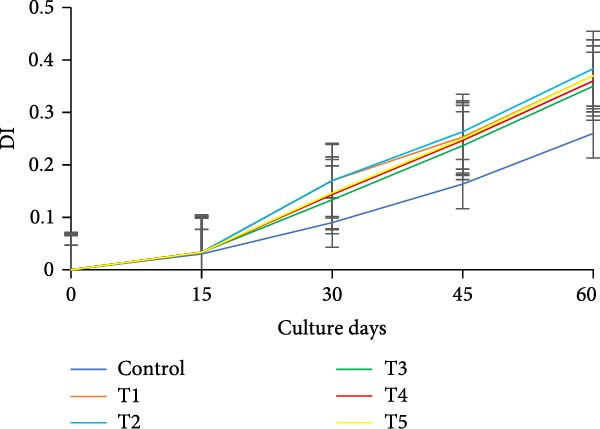
(d)

**Figure:**
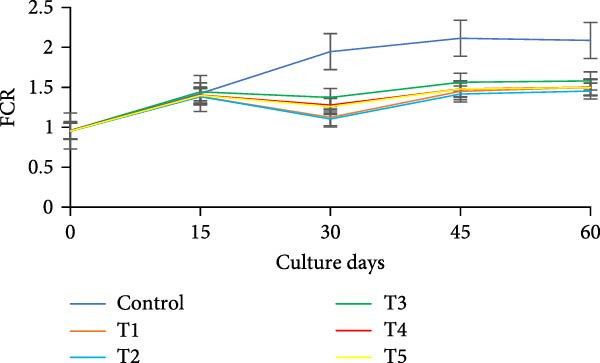
(e)

**Figure:**
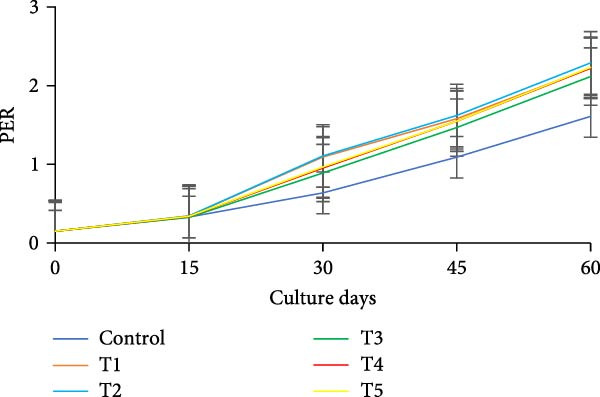
(f)

**Table 3:** Growth parameters during the 60‐day culture period.

Parameters	T1	T2	T3	T4	T5	Control
Initial weight (g/fish)	1.56 ± 0.003	1.57 ± 0.00	1.57 ± 0.00	1.57 ± 0.001	1.57 ± 0.00	1.57 ± 0.00
Final weight (g/fish)	23.73 ± 0.08	24.46 ± 0.14	22.56 ± 0.08	23.73 ± 0.17	23.83 ± 0.26	17.19 ± 1.00
Initial length (cm)	2.81 ± 0.003	2.82 ± 0.008	2.82 ± 0.008	2.83 ± 0.008	2.82 ± 0.008	2.83 ± 0.003
Final length (cm)	14.13 ± 0.12	14.33 ± 0.12	14.46 ± 0.14	15.63 ± 0.08	15.8 ± 0.05	10.16 ± 0.08
Total weight gain (g/fish)	9.94 ± 0.37^ab^	10.19 ± 0.18^a^	8.98 ± 0.16^c^	9.55 ± 0.14^bc^	9.60 ± 0.20^ab^	6.57 ± 0.55^d^
SGR (% per day)	1.15 ± 0.03^a^	1.15 ± 0.02^a^	1.09 ± 0.01^b^	1.12 ± 0.0^ab^	1.12 ± 0.01^ab^	0.96 ± 0.03^c^
DI	0.16 ± 0.00^ab^	0.17 ± 0.00^a^	0.15 ± 0.00^a^	0.15 ± 0.0^bc^	0.16 ± 0.00^abc^	0.10 ± 0.00^d^
FCR	1.28 ± 0.05^bc^	1.26 ± 0.04^c^	1.38 ± 0.03^c^	1.32 ± 0.02^bc^	1.31 ± 0.3^bc^	1.70 ± 0.10^a^
PER	1.07 ± 0.03^ab^	1.10 ± 0.01^a^	0.99 ± 0.01^c^	1.04 ± 0.01^bc^	1.04 ± 0.01^ab^	0.76 ± 0.05^d^
Survival rate (%)	93.3 ± 11.5^a^	96.6 ± 5.77^a^	96.6 ± 5.77^a^	93.3 ± 5.77^a^	93.3 ± 5.77^a^	90 ± 10^a^

*Note*: Values (means ± SE) in the same row with different superscripts differ significantly (*p*  < 0.05) between treatments.

### 3.3. Floc Formation and Structural Characteristics

Treatments receiving carbon inputs developed visible biofloc aggregates of varying densities, colors, and structures, while the CN group (no carbon or floc inoculum) showed minimal suspended solids and no visible floc formation (Table 4). As shown in Figure 4, floc parameters such as FV, FVI, FDI, porosity, and total solids varied significantly (*p*  < 0.05) among treatments. T5 (*E. crassipes* + jaggery) recorded the highest FV and porosity, followed by T2 (jaggery) and T4 (*E. crassipes*). These findings suggest that the combination of fibrous plant matter with a readily fermentable sugar source provided optimal conditions for microbial proliferation and organic aggregation, leading to higher‐quality flocs.

Figure 4Floc parameters of GIFT tilapia cultured during 60 days: (a) FV, (b) FVI, (c) FDI, (d) FP, (e) TSS, (f) TDS, and (g) TS.

**Figure:**
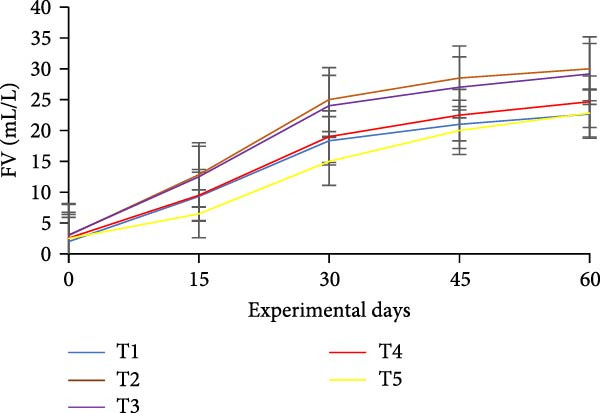
(a)

**Figure:**
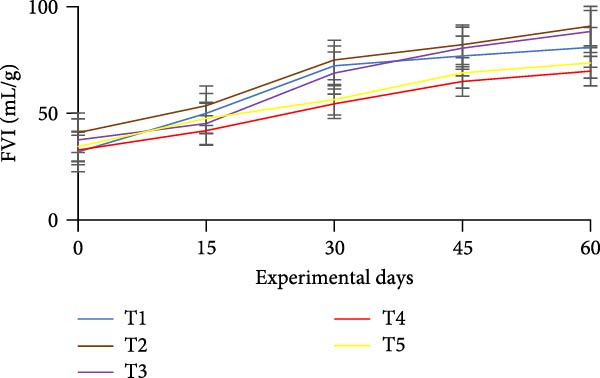
(b)

**Figure:**
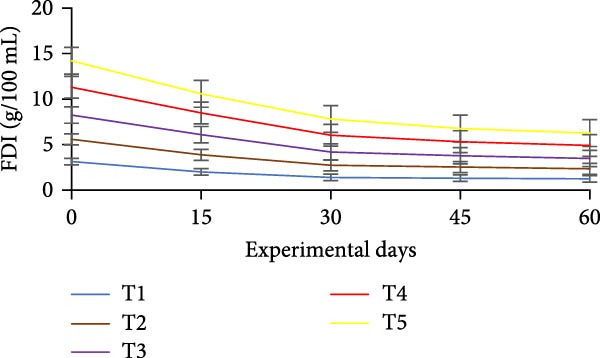
(c)

**Figure:**
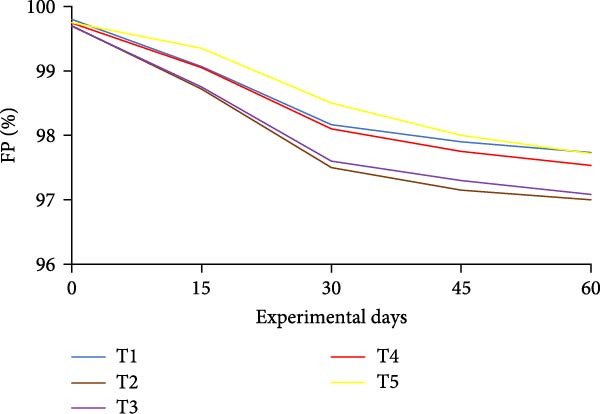
(d)

**Figure:**
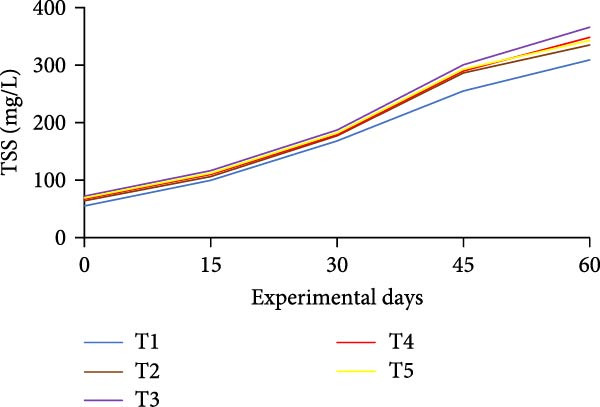
(e)

**Figure:**
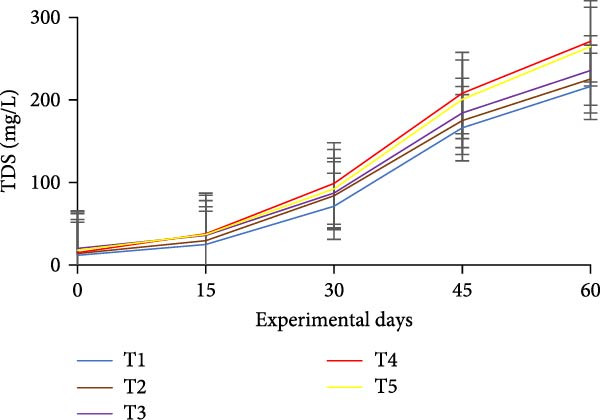
(f)

**Figure:**
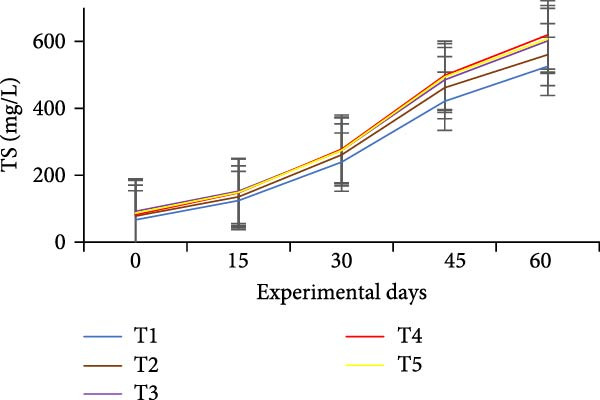
(g)

**Table 4:** Description of floc characteristics from different treatment tanks.

Treatment	Floc color	Floc size (µm)	Floc characteristics
T1	Middle brown (BS381C‐411)	95.40–392.00	Free flowing, grainy, and low uniformity
T2	Beaver (08‐B‐25)	175.28–443.88	Smooth texture, slurry‐like
T3	Black (18‐A‐14)	169.28–510.87	Smooth texture, slurry‐like, and denser
T4	Golden brown (BS2660‐3044)	192.38–510.87	Gritty texture, particulate slurry‐like
T5	Brown (04‐B‐29)	159.28–536.49	Smooth texture, slurry‐like, uniform.

**Figure 5:**
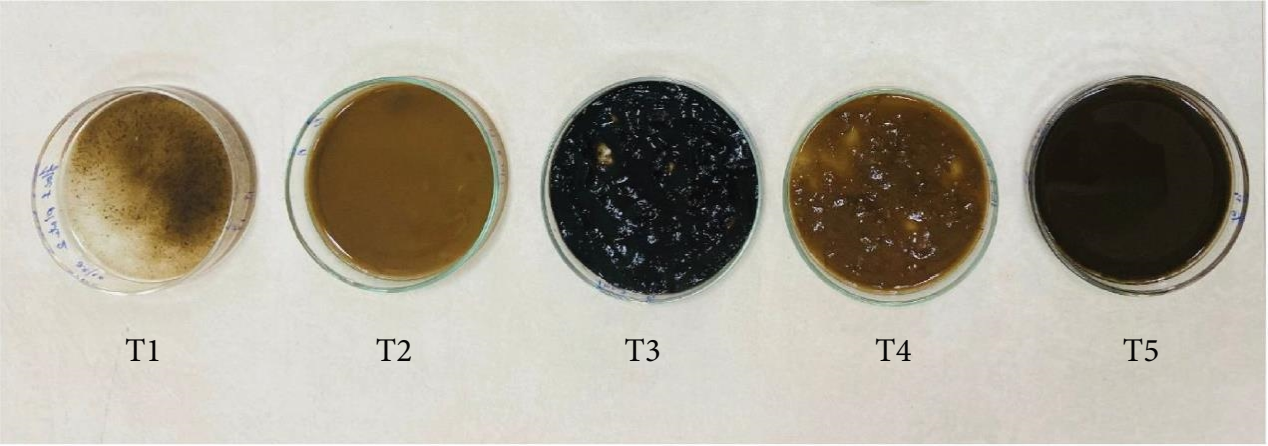
Morphological differences observed in experimental tanks across treatments using various carbon sources, highlighting floc density, coloration, and water clarity on Day 60.

Floc porosity and FVI reflect the ability of flocs to entrap water and nutrients, affecting settleability and microbial activity. The higher FVI observed in T5 and T2 indicates larger, less dense aggregates favorable for nutrient retention. On the other hand, lower FVI and porosity values in the CN confirm poor microbial colonization and floc development in the absence of carbon addition. The visible morphological differences among treatments are documented in Figure 5, where compact and dense flocs were observed in jaggery‐based and *E. crassipes*‐based treatments, while loose and irregular flocs were seen in the rice water and sugar groups. The CN tanks remained relatively clear, with little particulate matter.

To further characterize the microstructure of bioflocs formed using *E. crassipes*, light microscopy and scanning electron microscope (SEM) imaging were performed. As depicted in Figure 6, light microscopic images (Figure 6a,b) revealed progressive aggregation of *E. crassipes* particles into dense microbial matrices, especially in the presence of jaggery. SEM images (Figure 6c,d) showed a clear transition from the fibrous architecture of raw *E. crassipes* (Figure 6c) to a porous, irregular, and microbially colonized surface (Figure 6d), indicating effective biofilm formation and extracellular polymeric substance (EPS) accumulation. Collectively, the results demonstrate that *E. crassipes*, especially when combined with jaggery, is a promising low‐cost carbon source for biofloc development, offering physical structure and sufficient carbohydrate content to sustain microbial growth and improve floc quality.

Figure 6Morphological and structural features of bioflocs from *Eichhornia crassipes*‐based treatments: Light microscope images showing fine and dense floc structures (100×) at the beginning (a) and at the end (b) of the experiment, SEM images at ×500 magnification showing raw *E. crassipes* fibers (c) and microbially colonized biofloc matrix after culture (d).

**Figure:**
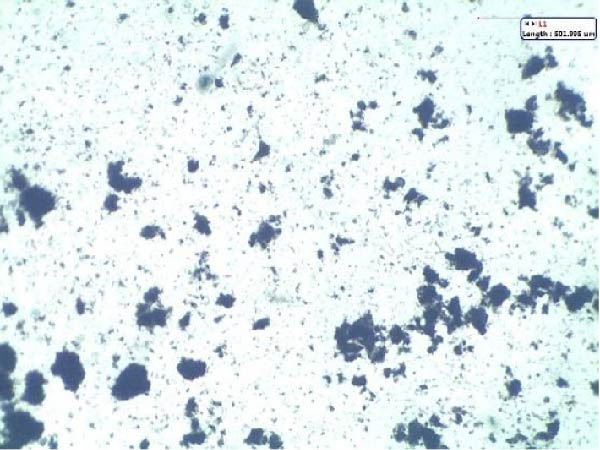
(a)

**Figure:**
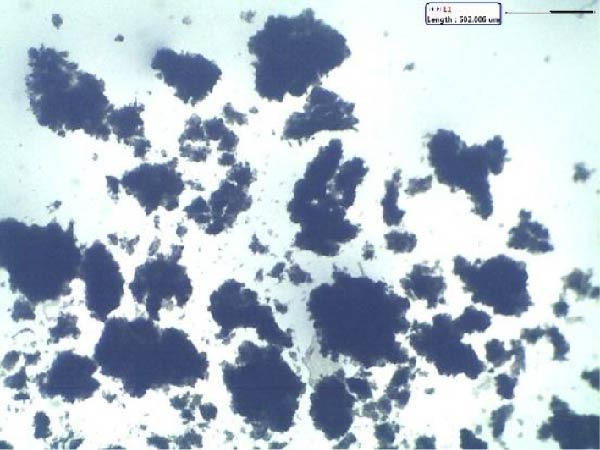
(b)

**Figure:**
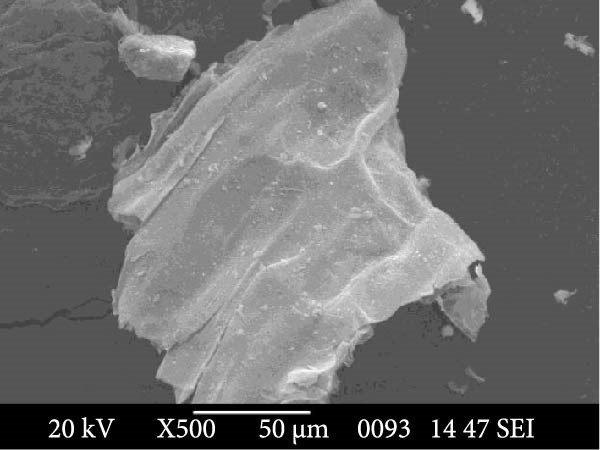
(c)

**Figure:**
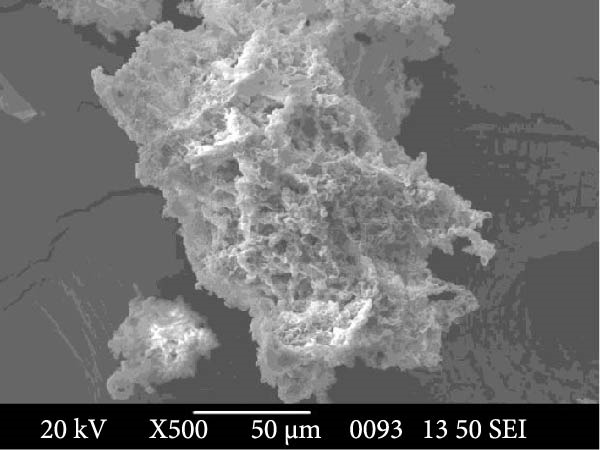
(d)

### 3.4. Bacterial Counts

**Figure 7:**
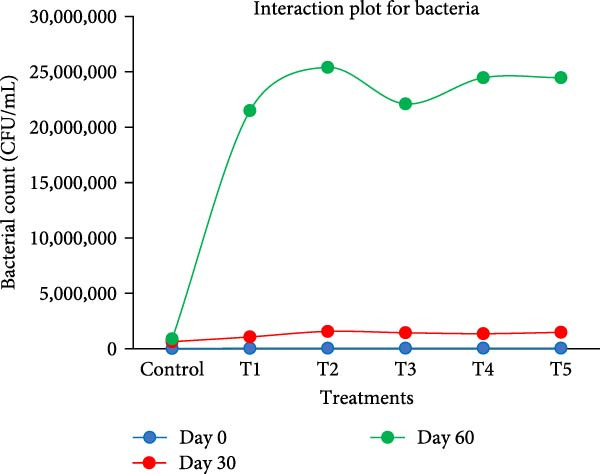
Total plate count of bacteria load from the experimental setups during the culture period.

At the beginning of the experiment, the highest microbial load was observed in T2 (54.67 ± 2.52 × 10^3^ CFU/mL), followed by T5 (50.67 ± 2.52 × 10^3^ CFU/mL), while the CN had the lowest count (26.67 ± 1.53 × 10^3^ CFU/mL). Statistical analysis revealed no significant differences between BFT treatments and CN (*p* > 0.05) (Figure 7). On the 30th day, similar trend in TPC was observed, with the highest in T2 (1566.67 ± 90.18 × 10^3^ CFU/mL), followed by T5 (1483.33 ± 70.24 × 10^3^ CFU/mL), while the CN (640.00 ± 40.00 × 10^3^ CFU/mL) remained the lowest (*p*  < 0.05). A significant difference in CN was observed (*p* < 0.05). The TPC of T1 was significant to all BFT treatments (*p*  < 0.05) except T4. However, T2, T3, T4, and T5 showed no significant differences among themselves. On the 60th day, T2 recorded the highest microbial load (2540.00 ± 30.00 × 10^4^ CFU/mL), followed by T4 and T5 (2446.67 ± 208.17 and 2446.67 ± 493.29 × 10^4^ CFU/mL, respectively), while the CN (900.00 ± 26.46 × 10^4^ CFU/mL) remained the lowest. At the end of culture days, the statistical analysis revealed no significant difference between T4 and T5 (*p*  > 0.05). However, T2 is significantly different from T4 and T5 (*p*  < 0.05).

### 3.5. Proximate Composition of Fish and Generated Floc

The result of the proximate composition of fish and floc samples after 60 days of experiment showed that different carbon sources significantly influenced the proximate composition of the GIFT Tilapia reared in biofloc systems and the flocs developed in the biofloc tanks (*p*  < 0.05) (Table 5). The protein content was significantly higher in T4 (*p*  < 0.05) followed by T5 and T1. The lipid content was significant (*p*  < 0.05) with maximum in T1 and T3 and ash content showed the highest in T3 (*p*  < 0.05). Except for Moisture and carbohydrate content, all other parameters, such as protein, lipid, and ash, were significantly lower (*p*  < 0.05) in CN than in the biofloc treatments. The protein and lipid content of biofloc exhibited a significant difference (*p* < 0.05), and the protein and lipid content were highest in T4 (*p* < 0.05). A significant difference (*p* < 0.05) was observed in the ash content among the treatments, with the maximum in T5 and the minimum in T2. The moisture content in the flocs varied from 90% to 92.5% and significantly higher carbohydrate was reported from T2 (*p* < 0.05).

**Table 5:** Proximate composition of fish and Floc during the 60‐day culture period.

Proximate composition	T1	T2	T3	T4	T5	Control
Fish						
Moisture	76.88 ± 0.04^b^	78.47 ± 0.01^a^	75.88 ± 0.41^bc^	74.65 ± 0.07^c^	76.41 ± 0.04^b^	79.22 ± 0.51^a^
Protein	17.85 ± 0.04^b^	15.51 ± 0.02^d^	16.14 ± 0.01^c^	18.23 ± 0.01^a^	17.88 ± 0.02^b^	15.13 ± 0.09^e^
Lipid	1.11 ± 0.01^a^	0.63 ± 0.02^d^	1.07 ± 0.02^a^	0.92 ± 0.01^b^	0.73 ± 0.02^c^	0.41 ± 0.01^e^
Ash	3.35 ± 0.01^d^	3.12 ± 0.01^e^	5.03 ± 0.03^a^	4.31 ± 0.01^b^	4.12 ± 0.01^c^	2.88 ± 0.02^f^
Carbohydrate	0.79 ± 0.08^b^	2.24 ± 0.01^a^	1.85 ± 0.37^ab^	1.87 ± 0.09^ab^	0.84 ± 0.08^b^	2.35 ± 0.41^a^
Flocs						
Moisture	91 ± 0.00^bc^	92.06 ± 0.07^a^	92 ± 0.00^a^	91.2 ± 0.06^b^	90.5 ± 0.29^c^	ND
Protein	1.87 ± 0.00^e^	2.37 ± 0.02^c^	2.94 ± 0.03^b^	3.41 ± 0.06^a^	3.33 ± 0.02^a^	ND
Lipid	0.27 ± 0.01^c^	0.08 ± 0.05^d^	0.45 ± 0.02^b^	0.66 ± 0.07^a^	0.34 ± 0.03^c^	ND
Ash	5.10 ± 0.01^a^	1.19 ± 0.00^d^	2.40 ± 0.01^c^	4.03 ± 0.03^b^	5.17 ± 0.01^a^	ND
Carbohydrate	1.74 ± 0.01^b^	4.29 ± 0.12^a^	2.19 ± 0.05^b^	0.68 ± 0.04^c^	0.64 ± 0.26^c^	ND

*Note*: Values (means ± SE) in the same row with different superscripts differ significantly (*p*  < 0.05) between treatments.

Abbreviation: ND, not detected.

## 4. Discussion

### 4.1. Water Quality Parameters

DO remained within suitable ranges for the survival and growth of GIFT tilapia throughout the trial [45]. From Day 30 onward, pH fluctuations emerged in the biofloc tanks, coinciding with marked changes in total alkalinity, whereas the CN maintained stable alkalinity as previously observed by Azim and Little [46]. These patterns indicate a loss of buffering capacity in BFT that warrants more frequent NaHCO_3_ additions. Because nitrification requires both oxygen and bicarbonate, persistent alkalinity drawdown is expected as microbial activity progresses [10]. Despite supplementation, alkalinity remained low, likely consumed during nitrification within the heterotrophic community, yet vigorous aeration kept DO adequate. Notably, treatment T5, despite exhibiting the lowest pH among BFT treatments, achieved the best growth and feed‐use efficiency, contrasting earlier observations [45]. The lower pH is consistent with intensified heterotrophic respiration and associated CO_2_ accumulation in biofloc systems [47, 48]. Water temperature (27–30°C) supported stable floc formation and an intermediate FVI, conditions compatible with favorable outcomes [13].

Ammonia dynamics followed the expected trajectory for newly established BFT. An initial increase occurred in all biofloc tanks, which can promote bacterial growth and jump‐start nitrification [35]. Targeting a moderate upper bound for ammonia (~3 mg/L) is known to favor nitrifier proliferation in recirculating and wastewater contexts [49, 50]. Subsequently, adding organic carbon reduced ammonia to permissible levels as nitrogen progressed along the pathway from NH_3_ to NO_2_^−^, NO_2_^−^ to NO_3_^−^, and ultimately to N_2_ [47, 51]. Carbon supplementation also enhanced heterotrophic assimilation of NH_3_ and limited NO_2_^−^ accumulation, particularly in T5 and T1. In parallel, *Eichhornia* likely provided surface area for nitrifiers, contributing to elevated NO_3_^−^ concentrations. Overall, NH_3_ and NO_2_^−^ levels were lower than many tilapia and shrimp BFT reports [21, 45, 52, 53], although ammonia sometimes exceeded values in comparable systems [34], plausibly due to reduced alkalinity and lower pH; NO_3_^−^ concentrations were higher than in several prior studies [21, 31, 34, 53]. Taken together, these data indicate that incorporating *E. crassipes* into BFT can improve overall water quality.

### 4.2. Growth Parameters

During the early phase, growth metrics were similar across treatments, likely reflecting the common basal diet and initial, limited floc availability. As biofloc matured, fish in BFT tanks plausibly consumed flocs in addition to the basal feed, which was absent in CN tanks, supporting progressive gains. Prior BFT studies indicate that floc presence enhances growth through continuous microbial protein and associated nutrients [54–56]. The microbial consortium proliferates via ammonia assimilation and aggregation, creating edible flocs [17, 57, 58]. These flocs provide proteins, minerals, vitamins, selected fatty acids, and carotenoids, and can modulate the gut microbiome, thereby acting as a supplementary feed and functional prebiotic matrix [17, 55, 56]. Final weight and PER did not differ significantly among carbon sources, consistent with the view that biofloc nutritional contribution depends as much on digestive assimilation as on nominal composition [59]. GIFT tilapia efficiently utilized biofloc generated with *E. crassipes*, yielding growth comparable to conventional carbon inputs. Similar parity among carbon sources was reported in tilapia and common carp using sugar, molasses, and cassava starch [21, 60]. Conversely, some studies observed higher weight gain with starch‐ or wheat‐based biofloc versus molasses/cassava [46, 61], underscoring species‐ and context‐specific responses. Feed conversion ratios (FCR) in the present study were lower than those reported by Komara et al. [34], although several trials document FCR >1.5 [46, 62–64].

Beyond growth, *E. crassipes*‐derived biofloc may confer health benefits. The macrophyte provides nutrients and vitamins that support microbial communities and farmed fish [65], including amino acids [66] and antioxidant/phenolic compounds [67]. Biofloc integrated with *E. crassipes* has been associated with improved immunity and disease resistance in tilapia [34], and vitamin C within *E. crassipes* can bolster antioxidant defenses and reduce stress [66]. Studies also report anti‐stress effects and better performance in GIFT tilapia reared with *E. crassipes*‐enriched biofloc [68]. *E. crassipes* extracts enhanced innate immunity and pathogen resistance in *Macrobrachium rosenbergii* [69] showing protection against S*treptococcus iniae* [70]. Collectively, these findings suggest that *E. crassipes*‐based floc functions as a supplementary, health‐promoting feed component while maintaining growth parity with conventional carbon sources.

### 4.3. Floc Parameters

FV, porosity, FDI, FVI, and solids (TS, TDS, TSS) are standard descriptors of biofloc quantity and quality. In this study, all BFT treatments showed significantly higher FV, TS, TSS, and TDS than the CN, consistent with previous reports [20, 34]. Intensive aeration and supplemental carbon drive the aggregation of uneaten feed and feces into microbial flocs, processes that are minimal in non‑BFT CNs, aligning with tilapia BFT literature [45, 46, 71–73].

Measured FV and TSS across BFT treatments were within finfish culture benchmarks (FV 25–50 mL/L; TSS < 500 mg/L) [74, 75]. FV was lower in T5 than in T4, which may reflect a synergistic effect when *E. crassipes* is combined with jaggery. Reduced FV implies smaller, more porous flocs [76]. Although smaller particles can slow nitrification due to uneven distributions of ammonia‑oxidizing and nitrite‑oxidizing bacteria across size classes [35, 77, 78], our results indicate that nitrification performance converged among carbon sources by the end of the trial. Floc metrics are also influenced by grazing. Floc consumption depends on floc size/density and fish traits (species, body size, feeding behavior) [13, 79]. Tilapia’s filter‑feeding can reduce suspended particulates and improve water quality via direct biofloc consumption [46, 79, 80]. FVI served as a proxy for settleability/floc age: lower FVI indicates better settleability and system efficiency. Improved FVI and settleability were observed in *E. crassipes* + jaggery BFT. The reference FVI range of 40–60 is considered suitable for sludge settling [13]. In our study, *E. crassipes*‐based BFT fell within the standard range, with other treatments comparable to benchmarks and lower than some reports [76], consistent with tilapia’s ability to utilize suspended flocs under semi‑bottom conditions.

Reported FDI values in activated sludge systems (~1.39 and 1.02 g/cm^3^) provide context [40, 76]. A higher FDI here indicates effective aggregation and denser flocs. We also noted an inverse relationship between porosity and FV: lower FV coincided with smaller, more porous flocs, whereas larger FV corresponded to bulkier, more compact aggregates; observed porosity was higher than in some earlier works [76, 77]. Beyond physical metrics, *E. crassipes* is a utilizable resource for multiple cultured species, including rohu, Nile tilapia, and siamese gourami [34, 81–83]. Building on this literature, the present study is, to our knowledge, the first to characterize floc properties with *E. crassipes* as a carbon source in BFT. *E. crassipes*–based BFT yielded the lowest FV with TSS within optimal limits, supporting its use as an efficient alternative to conventional carbon sources.

### 4.4. Floc Morphology

Floc appearance changed predictably over time. During the first days, water in all BFT tanks was greenish, shifted to yellowish‑brown by week one, and progressively darkened to brown/black toward the end of culture. These transitions reflect a shift from photoautotrophic contributions to heterotrophic dominance driven by carbon dosing, aeration, and C:N management. Brown flocs are typically microbe‑dominated with limited microalgal contribution under low‑to‑moderate carbon regimes [48], and their spectral characteristics have been linked to pigmented heterotrophic bacteria [84]. Tilapia readily consume biofloc irrespective of size [80]. Floc size influenced nutritional profile and performance. Larger particles (>100 *μ* m) tended to have higher protein (~27.8%) and lipid (~7.5%), whereas smaller fractions (<48 *μ* m) were richer in essential amino acids [80]. Despite lower crude protein, smaller flocs have been associated with better growth and reduced gill damage during prolonged tilapia culture [84], and reductions in floc size improved growth and feeding efficiency in *Ompok bimaculatus* fry, likely via enhanced ingestion [85]. Reported size spectra vary among species and systems [80, 86], but collectively, floc architecture governs surface area for microbial attachment, nutrient adsorption, and nitrogen retention [80]. In the present study, superior floc formation in the *E. crassipes* + jaggery treatment is consistent with reports that complex carbon substrates enhance floc composition and microbial activity [20, 22]. Observed color and size trajectories therefore align with a heterotroph‑dominated, nutritionally functional biofloc that remains readily ingestible by tilapia.

### 4.5. Bacterial Count

The results indicate that all biofloc treatments significantly enhanced microbial load compared to the CN, suggesting that biofloc systems promote microbial proliferation. T2 consistently exhibited the highest microbial load, possibly due to an optimal carbon/nitrogen ratio and nutrient availability. The increasing trend from Days 0–60 aligns with biofloc maturation, where microbial communities stabilize over time. The bacterial count increased steadily over time, reflecting active bacterial proliferation in all biofloc tanks, suggesting the efficiency of carbon sources [51, 60, 76]. This suggests that BFT enhances microbial activity, which may contribute to improved water quality and tilapia growth [87, 88]. The bacterial count in biofloc may increase when jaggery is used as a carbon source. Researchers suggest that jaggery efficiently promotes microbial load [20, 56, 89]. According to Elayaraja et al. [90], jaggery‐based BFT with C:N ratio of 20 provides better growth performance, enhanced antioxidant capacity, and disease resistance to *A. hydrophila*. Also, it favored the growth of bacteria, notably *Bacillus kochii*, *Virgibacillus* spp [91]. The proportional growth in T5 may be the synergistic effect of jaggery and *E. crassipes*. However, *E. crassipes* alone exhibited similar growth to T5. This may be attributed to the presence of cellulose in *E. crassipes*, which supports bacterial proliferation by providing both nutrients and a supportive matrix. A study by plant cellulose improves bacterial diversity in biofloc systems, particularly by enhancing the ammonia‐oxidizing bacteria and increasing the relative abundance of *Alphaproteobacteria* [88]. *Rhodobacteraceae*, a prominent family within *Alphaproteobacteria*, consistently dominates microbial communities in biofloc systems and plays key ecological roles in system stability and nutrient cycling [92].

### 4.6. Proximate Composition of Fish and Generated Floc

Fish reared in BFT exhibited higher whole‑body protein, lipid, and ash than those in clear water, indicating a compositional shift associated with access to biofloc [17, 24, 56, 93–95]. The generated flocs likely contributed digestible microbial biomass and micronutrients, driving the observed differences. Carbon source influenced proximate outcomes, as reported previously [56, 76, 96]. In our trial, treatments using *E. crassipes* (T4 and T5) showed higher protein, plausibly reflecting the plant’s amino acid and vitamin profile. *E. crassipes* contains essential amino acids such as isoleucine, lysine, serine, tyrosine, tryptophan, valine, leucine, histidine, methionine, glutamic, and aspartic acids [66, 97] and is particularly rich in vitamin C, with notable vitamin A content relative to common feed grains [66]. Not all studies detect protein shifts among carbon sources [24, 93], underscoring context‑specific responses and the role of digestive assimilation. Lipid content was elevated in T1 and T3 versus other BFT groups. Although bioflocs are typically low in lipids, their nutritional value is enhanced by microbial fatty acids and associated compounds [46, 98]. Antioxidant capacity derived from *E. crassipe*s, linked to polyphenols and flavonoids, may further support tissue composition and stress resilience [99]. Elemental analyses indicate stems contain more Na, Mg, Ca, Mn, and Fe than leaves, while stem structural carbohydrates are lower [100].

Beyond proximate composition, *E. crassipes* provides bioactive and therapeutic compounds with reported antibacterial, antifungal, and other activities, highlighting its broader utility [101]. Nutritionally, it offers protein for livestock and aquaculture feeds, supporting substitution of costlier ingredients where feasible [65, 102]. As a noxious macrophyte, its use as a feed resource can also be guided by availability and economics [103]. Leaf fractions tend to be protein‑ and fiber‑rich, whereas stems contribute more carbohydrates and crude lipids [101]. Collectively, these attributes support *E. crassipes* as an alternative input in BFT that can favorably influence fish body composition while supplying functional nutrients.

## 5. Conclusion

This is the first study in India to demonstrate the use of the aquatic invasive water hyacinth macrophyte, *E. crassipes*, as a carbon source in biofloc systems. Low‐cost, globally available *E. crassipes* can easily replace other conventional carbon sources in BFT due to its higher carbohydrate, lipid, and protein with rich antioxidants and vitamins resulting in improved floc composition. The study concludes that supplementation of *E. crassipes* alone can offer excellent growth, survival, water quality, and improved metabolism. However, the mixture of *E. crassipes* and jaggery‐based biofloc can improve the floc characteristics when compared to *E. crassipes*‐based BFT. The study concludes that the aquatic weed *E. crassipes* alone may be more efficient for enhancing growth and survival compared to the mixture‐based system, which would increase production costs. The application of the aquatic weed can reduce environmental and ecological impacts while enhancing carbon availability in BFT, effectively recycling waste into wealth and supporting aquaculture with improved growth and nutrition. This innovative research focuses on sustainable green living with reduced cost of production for fish farmers. In conclusion, *E. crassipes* can be used as an efficient, zero‐cost, easily available carbon source with improved nutrition and survival over any other conventional carbon sources. More research is required to identify the effective part of *E. crassipes* as a carbon source, dosage, and stocking density trials.

## Conflicts of Interest

The authors declare no conflicts of interest.

## Author Contributions

**Megha Lovejan:** conceptualization, data curation, formal analysis, methodology, writing – original draft, validation, writing – review and editing. **K. M. Mujeeb Rahiman**: conceptualization, supervision, methodology, writing – review and editing, validation. **C. G. Joshy**: formal analysis, data curation. **R. Vipina**: formal analysis, validation**. Remy Ntakirutimana:** formal analysis, software, data curation, writing – review and editing.

## Funding

This work was supported by the Government of Kerala (Grant EG/2018/00929247).

## Data Availability

The data will be made available upon request.
